# The characterisation of overweight and obese women who are under reporting energy intake during pregnancy

**DOI:** 10.1186/s12884-018-1826-x

**Published:** 2018-06-01

**Authors:** L. J. Moran, S. A. McNaughton, Z. Sui, C. Cramp, A. R. Deussen, R. M. Grivell, J. M. Dodd

**Affiliations:** 1 The Robinson Research Institute, Discipline of Obstetrics and Gynaecology, School of Paediatrics and Reproductive Health, The University of Adelaide, Adelaide, VIC 3168 Australia; 2 Monash Centre for Health Research Implementation, School of Public Health and Preventive Medicine, Monash University, Clayton, Australia; 3 Institute for Physical Activity and Nutrition (IPAN), Deakin University, Geelong, VIC Australia; 4 Department of Perinatal Medicine, Women’s & Babies Division, Women’s and Children’s Hospital, North Adelaide, Adelaide, Australia

**Keywords:** Under reporting of energy, Diet, Pregnancy, Obesity, Overweight

## Abstract

**Background:**

Misreporting of energy intake is common and can contribute to biased estimates of the relationship between diet and disease. Energy intake misreporting is poorly understood in pregnancy and there is limited research assessing characteristics of women who misreport energy intake or changes in misreporting of energy intake across pregnancy.

**Methods:**

An observational study in *n* = 945 overweight or obese pregnant women receiving standard antenatal care who participated in the LIMIT randomised trial. Diet, physical activity, psychological factors, body image satisfaction and dieting behaviour were assessed at trial entry (10–20 weeks gestation) and 36 weeks gestation. Energy misreporting status was assessed through the ratio of daily energy intake over basal metabolic rate. Logistic regression analyses were conducted with the dependent variable of under reporting of energy intake at study entry or 36 weeks in separate analysis.

**Results:**

At study entry and 36 weeks, women were classified as under reporters (38 vs 49.4%), adequate reporters (59.7 vs 49.8%) or over reporters of energy intake (2.3 vs 0.8%) respectively. The prevalence of under reporting energy intake at 36 weeks was higher than at study entry (early pregnancy). Body mass index (BMI) at study entry and 36 weeks and socioeconomic status, dieting behaviour and risk of depression at 36 weeks were independent predictors of under reporting of energy intake.

**Conclusions:**

Under reporting of energy intake was present in over a third of overweight and obese pregnant women and was higher in late compared to early pregnancy. Characteristics such as BMI, socioeconomic status, past dieting behaviour and risk of depression may aid in identifying women who either require support in accurate recording of food intake or attention for improving diet quality. Results were unable to distinguish whether under reporting reflects misreporting or a true restriction of dietary intake.

**Trial registration:**

Australian and New Zealand Clinical Trials Registry ACTRN12607000161426, registered 9/3/2007.

**Electronic supplementary material:**

The online version of this article (10.1186/s12884-018-1826-x) contains supplementary material, which is available to authorized users.

## Background

There are recognised limitations to the measurement of dietary intake in clinical or epidemiological research. Commonly used tools including food frequency questionnaires (FFQ), 24-h dietary recalls and dietary records can result in misreporting (low or high reporting) of dietary intake in comparison with gold-standard measures such as doubly labelled water and 24-h urinary nitrogen excretion [1]. The prevalence of misreporting varies but is estimated at ~ 30% for under reporting of energy intake and ~ 10% for over reporting of energy intake in the general adult population [2]. Misreporting also varies depending on the dietary assessment tool used with a lower level of low energy reporting for multiple 24-h recalls compared with a FFQ [3]. Misreporting is related to characteristics including adiposity, socioeconomic status and education, age, gender, psychological status such as depression or poor body image, or health-related activities such as smoking or dieting [2, 4]. Misreporting of energy intake could reflect intentional under recording which can be related to social desirability bias. In particular, low energy reporting may result from biasing of reported intake towards foods deemed more appropriate [4, 5] with a lower intake of unhealthier foods high in fat and sugar or unhealthy eating habits resulting in an overall lower diet quality [4]. It may also be related to failure to record accurate food intake due to recall bias or memory lapses, poor awareness of quantities or types of foods eaten [2], the inconvenience of reporting, or reporting an incomplete or simplified version of what is consumed secondary to inaccurate portion size estimation [6].

The misreporting of dietary intake, particularly if systematic and non-random, can result in incorrect assessments of the relationships between dietary components and clinical outcomes. The common under reporting of energy intake or specific dietary components in obesity may also result in a specific bias in studies investigating the relationship between the aetiology and consequences of obesity. Stronger associations between diet, obesity or obesity-associated health conditions or biomarkers of diet or obesity-related health have been previously reported in adequate compared to under reporters of energy intake [7, 8].

Ensuring adequate dietary intake during pregnancy is crucial for optimising maternal, fetal, and infant outcomes [9, 10]. This is important with regards to ensuring micronutrient adequacy and preventing excess energy and macronutrient intake. The identification of women who misreport energy intake in pregnancy is important in identifying any associations between maternal antenatal diet and subsequent health outcomes. This is also relevant in overweight and obese pregnant women as they are more likely to have a higher prevalence of under reporting of energy intake [11], and an increased risk of adverse pregnancy and birth complications. [12] Furthermore, children born to women who are overweight or obese have a higher prevalence of childhood and adult obesity, and obesity-related conditions [13, 14]. However, there is limited literature examining the characteristics of pregnant women who misreport energy intake. While psychological characteristics are associated with under-reporting in the general population [4, 15], there is minimal examination of depression [16] and no examination of the contribution of anxiety, body image status or dieting behaviour to misreporting in pregnancy. There is also limited and contradictory literature examining changes in misreporting status across pregnancy [17, 18], it being unclear if there is a true reduction in energy intake related dietary restriction or to a higher level of energy misreporting.

The aim of this study was to perform a comprehensive assessment of energy misreporting status across two time-points in pregnancy (early and late pregnancy) in a large population of overweight and obese women. Specifically, we aimed to assess the demographic, behavioural and psychological characteristics of overweight and obese pregnant women who misreported energy intake or who had changes in energy misreporting status across pregnancy.

## Methods

### Study design and population

This study involved women who participated in the LIMIT randomised controlled trial, and who were randomised to receive Standard Antenatal Care. The detailed methodology and primary trial findings have been reported [19]. Briefly, *n* = 2122 women with a body mass index (BMI) ≥25 kg/m^2^, with a singleton pregnancy between 10 and 20 weeks gestation (defined as trial entry) and no current diagnosis of diabetes were recruited from the three main maternity hospitals in metropolitan Adelaide, South Australia. The ethics committee at each collaborating hospital independently approved the study protocol (Women’s and Children’s Hospital (Adelaide, South Australia); Flinders Medical Centre (Adelaide, South Australia); Lyell McEwin Health Service (Adelaide, South Australia); The Queen Elizabeth Hospital (Adelaide, South Australia), and eligible women provided written informed consent. At the time of study entry, women were stratified according to the following domains; parity (0 versus 1 or more), BMI at antenatal booking visit (25.1–29.9 kg/m^2^ versus > 30 kg/m^2^) and collaborating centre. The LIMIT trial was registered at the Australian and New Zealand Clinical Trials Registry (ACTRN12607000161426).

### Outcomes

#### Demographics and maternal and obstetric outcomes

Baseline demographic details were collected including parity, age, ethnicity, smoking, and past obstetric, medical and family history. Socioeconomic index for area of disadvantage score (SEIFA) [20] was calculated from postcode of residence, with quintile 1 indicating the most disadvantaged women. Women had their weight and height measured by research staff at their first trimester antenatal visit (median 14 weeks gestation) [19] using calibrated scales and stadiometer and weight measured again at 36 weeks gestation. BMI was calculated as their weight divided by the square of their height. Gestational weight gain was calculated as the change in their weight between measurements. BMI was categorised as overweight (25 to 29.9 kg/m^2^), obese class I (30–34.99 kg/m^2^), class II (35–39.99 kg/m^2^) or class III (≥40 kg/m^2^) according to World Health Organization guidelines [21] and gestational weight gain was classified as below (inadequate), within (adequate) or above (excessive) according to the Institute of Medicine guidelines [22] of 7–11.5 kg for overweight and 5–9 kg for obese women for total weight gain.

#### Risk of depression, anxiety symptoms and body image

At study entry and 36 weeks gestation, women were asked to complete questionnaires assessing risk of depression and anxiety as previously reported [23], in addition to questionnaires assessing weight and shape satisfaction. Risk of depression was measured using the Edinburgh Postnatal Depression Scale (EPDS), with increased risk of depression defined as a score of ≥12 [24]. Maternal anxiety symptoms were assessed using the Spielberger State-Trait Inventory Self Evaluation Questionnaire (STAI), comprising of 6 questions, with scores below 15 considered to be within the normal range [25, 26].

At study entry women were asked to complete questionnaires relating to their visual perception of body image in which standard silhouettes were presented corresponding to different BMI categories (Stunkard Figure Rating Scale) [27]. Women were asked to indicate the silhouette that most closely resembled how they usually look (prior to pregnancy), and that which represented how they would like to look. This produced a measure of current size, desired size and a discrepancy score (desired-measured) as a measure of body dissatisfaction. In addition, women were asked to indicate the number of times in the previous year they had attempted to limit the amount of food eaten in order to lose weight (responses never, 1 to 10 times or more than 10 times) and their satisfaction with their weight and body shape (classified as satisfied if they indicated markedly satisfied, moderately satisfied or slightly satisfied versus not at all satisfied) as previously described [28].

#### Dietary intake, physical activity and energy reporting status

At trial entry and 36 weeks, dietary intake was assessed with the Harvard Semi-quantitative FFQ (The Willett Questionnaire). The reference period for the FFQ was the past 12 months (for trial entry questionnaires) and for the time period from week 28–36 (for the 36 week questionnaire due to the administration of an additional FFQ at 28 weeks). The Willett questionnaire was developed in 1985 in the USA to measure the daily intake of nutrients from 126 food items, with an indication of standard portion size, divided into seven food groups [29], and has been validated for use during pregnancy [30], and in an Australian pregnancy setting [31]. Questions were asked about the relative frequency of consumption of specific food items, use of supplements, cooking methods used and addition of sugar to foods. An open-ended question allowed record of consumption of other foods, which were then categorised by the study investigators. Daily energy, macronutrient and micronutrient intakes were estimated by multiplying frequency responses by the nutrient compositions of the specified portion size of each food item according to Australian food composition tables [32], reflecting standard food fortification with both folate and iodine, utilising the Food Works Nutrient Analysis Software Package (FoodWorks, v.7 Professional; Xyris Software 2012; Australia). Glycemic index values for each food using glucose as the reference standard were obtained from published international [33] and online (http://www.glycemicindex.com/) glycemic index tables according to a prior published protocol [34]. Dietary glycaemic index was determined as the sum of the glycaemic index for all carbohydrates consumed in the diet, with a proportional weighting to account for the relative contribution of each food. Dietary glycemic load was calculated by multiplying the glycemic index of each food with the amount of carbohydrate per serve (from Australian food composition tables [32]) with the overall glycemic load summed from all foods. Diet quality was assessed through the 2005 Healthy Eating Index (HEI) as previously described calculated on an energy-adjusted density [35]. The maximum score is 100 with a HEI score above 80 considered good, a score between 50 and 80 needing improvement and scores below 50 considered poor. If missing data exceeded 25% the questionnaire was excluded from the analysis. At trial entry and 36 weeks total, work, housework, commuting and leisure-time physical activity in metabolic equivalents (MET)/minute/week were assessed with the Short Questionnaire to Assess Health-enhancing physical activity (SQUASH) as previously described [35].

Adequate reporting of energy intake was determined by Goldberg’s method using the ratio of daily energy intake to estimated basal metabolic rate (BMR). BMR was calculated for each woman using Schofield equations using age, gender and weight [36] as previously reported in pregnancy [37, 38]. The extra energy requirement for different trimesters was then added to BMR according to National Health and Medical Council reference values (1.4 MJ/day for the 2nd and 1.9 MJ/day for the 3rd trimester) [39]. Under reporters of energy intake, adequate reporters and over reporters of energy intake were classified as an energy intake of < 0.9, 0.9 to 2.1 and > 2.1 BMR respectively as this has been previously used to assess misreporting in pregnant populations [37, 38, 40, 41].

### Statistical analysis

Two-tailed statistical analyses were performed using Stata (Stata/IC 13.1 for Windows 2014) with statistical significance set at a *P* value of < 0.05. Data are presented as mean ± standard deviation (SD) except where indicated. Data transformations were not used to correct for any departures from normality, since the sample size was sufficient for the central limit theorem to apply [42]. Cross-sectional data were assessed using t test or chi square test and comparisons between time points were assessed using Wilcoxon test for non-parametric and paired t test for parametric data with energy reporting status as the between subject factor. Logistic regression analyses were conducted with the dependent variable of under reporting of energy intake at study entry or 36 weeks in separate analysis. Independent variables were selected based on hypothesis testing and association with under reporting of energy intake on univariate analysis (*p* < 0.2) with the final model constructed with simultaneous entry of preselected predictors. Regression models were constructed to avoid collinearity and assessed for standard assumptions.

## Results

### Participant characteristics

This study comprises *n* = 945 women randomised to the routine care group of LIMIT (age 29.6 ± 5.4 years; weight 87.7 ± 17.1 kg and BMI 32.3 ± 5.8 kg/m^2^). Of these women, data were available at 36 weeks for *n* = 508 women. For the total sample, women were classified as under reporters of energy intake [359/945 (38.0%) at study entry; and 251/508 (49.4%) at 36 weeks respectively], adequate reporters of energy intake [564/945 (59.7%) and 253/508 (49.8%)] or over reporters of energy intake [22/945 (2.3%) and 4/508 (0.8%)]. These proportions were numerically similar when assessing the completers analyses only (Table 1). As there were only a small number of women who over reported their energy intake at each time point, further statistical analysis with this subgroup was not possible and they were excluded from all further analyses.

**Table 1:** Participant characteristics according to energy reporting status

Outcomes		10–20 weeks (total sample, *n* = 923)			10–20 weeks (completers, *n* = 497)			36 weeks (*n* = 504)		
		UR *N* = 359 (38.9%)	AR *N* = 564 (61.1%)	*P*	UR *N* = 188 37.8%)	AR *N* = 309 (62.2%)	*P*	UR *N* = 251 (49.8%)	AR *N* = 253 (50.2%)	*P*
EI/BMR	mean ± SD	0.71 ± 0.15	1.25 ± 0.26	< 0.001	0.72 ± 0.13	1.25 ± 0.27	< 0.001	0.68 ± 0.14	1.21 ± 0.26	< 0.001
Physical activity (MET/min/wk)	mean ± SD	6838.3 ± 4309.1	7661.5 ± 198.1	0.008	6877.8 ± 4073.6	7555.7 ± 4099.4	0.074	5489.0 ± 3889.7	5568.3 ± 3680.9	0.816
Energy intake (kJ)	mean ± SD	5707.5 ± 1215.0	9726.3 ± 2082.2	< 0.001	5776.8 ± 1142.9	9673.7 ± 2101.0	< 0.001	5986.5 ± 1258.9	10,400.8 ± 2202.9	< 0.001
HEI total	mean ± SD	71.5 ± 7.8	73.0 ± 7.0	0.002	72.2 ± 7.7	73.4 ± 6.9	0.085	70.8 ± 7.9	71.5 ± 7.5	0.319
Age (years)	mean ± SD	28.5 ± 5.5	30.2 ± 5.2	< 0.001	28.9 ± 5.1	30.4 ± 29.9	0.001	29.4 ± 5.2	30.4 ± 5.0	0.029
	< 20 years	16 (4.5%)	10 (1.8%)	0.002	4 (2.1%)	5 (1.6%)	0.241	5 (2.0%)	4 (1.6%)	0.505
	20- < 30 years	193 (53.8%)	255 (45.2%)		96 (51.1%)	134 (43.4%)		123 (49.0%)	109 (43.1%)	
	30- < 40 years	141 (39.3%)	276 (48.9%)		85 (45.2%)	159 (51.5%)		117 (46.6%)	131 (51.8%)	
	≥40 years	9 (2.5%)	23 (4.1%)		3 (1.6%)	11 (3.6%)		6 (2.4%)	9 (3.6%)	
Weight (kg)	mean ± SD	92.7 ± 18.0	84.7 ± 15.8	< 0.001	91.7 ± 17.6	83.3 ± 14.5	< 0.001	99.4 ± 17.4	93.4 ± 13.3	< 0.001
BMI (kg/m^2^)	mean ± SD	33.9 ± 6.1	31.3 ± 5.4	< 0.001	33.5 ± 6.2	30.8 ± 5.0	< 0.001	36.7 ± 5.8	34.3 ± 4.6	< 0.001
	Overweight	119 (33.1%)	287 (50.9%)	< 0.001	65 (34.6%)	162 (52.4%)	< 0.001	20 (8.0%)	34 (13.4%)	< 0.001
	Obese class I	98 (27.3%)	163 (28.9%)		56 (29.8%)	96 (31.1%)		97 (38.6%)	129 (51.0%)	
	Obese class II	81 (22.6%)	70 (12.4%)		38 (20.2%)	34 (11.0%)		72 (28.7%)	63 (24.9%)	
	Obese class III	61 (17.0%)	44 (7.8%)		29 (15.4%)	17 (5.5%)		62 (24.7%)	27 (10.7%)	
Parity	0	155 (43.2%)	219 (38.8%)	0.190	84 (44.7%)	130 (42.1%)	0.569	115 (45.8%)	102 (40.3%)	0.212
	≥1	204 (56.8%)	345 (61.2%)		104 (55.3%)	179 (57.9%)		136 (54.2%)	151 (59.7%)	
Smoking	No	308 (88.0%)	498 (90.5%)	0.224	165 (89.7%)	286 (94.4%)	0.054	25 (89.8%)	11 (95.6%)	0.014
	Yes	42 (12.0%)	52 (9.5%)		19 (10.3%)	17 (5.6%)		221 (10.2%)	237 (4.4%)	
SEIFA	Quintile 1	105 (29.3%)	164 (29.1%)	0.194	48 (25.5%)	72 (23.3%)	0.382	70 (27.9%)	50 (19.8%)	0.056
	Quintile 2	102 (28.5%)	124 (22.0%)		58 (30.9%)	75 (24.3%)		73 (29.1%)	64 (25.3%)	
	Quintile 3	48 (13.4%)	92 (16.3%)		29 (15.4%)	54 (17.5%)		41 (16.3%)	43 (17.0%)	
	Quintile 4	51 (14.2%)	93 (16.5%)		28 (14.9%)	54 (17.5%)		35 (13.9%)	48 (19.0%)	
	Quintile 5	52 (14.5%)	91 (16.1%)		25 (13.3%)	54 (17.5%)		32 (12.7%)	48 (19.0%)	
Ethnicity	Caucasian	330 (91.9%)	515 (91.3%)	0.745	176 (93.6%)	288 (93.2%)	0.858	233 (92.8%)	236 (93.3%)	0.157
	Non-Caucasian	29 (9.1%)	49 (8.7%)		12 (6.4%)	21 (6.8%)		18 (7.2%)	17 (6.7%)	

Data are presented as mean ± SD or proportions and were analysed by independent t test or chi squared tests

Over reporters were excluded from analysis

*AR* Adequate reporter, *ATSI* Aboriginal or Torres Strait Islander, *EI/BMR* energy intake: basal metabolic rate, *GDM* gestational diabetes mellitus; *HEI* Healthy Eating Index, *MET* Metabolic equivalent, *SD* Standard deviation, *SEIFA* Socioeconomic indices for areas disadvantage score South Asian: Indian/Pakistan/Sri Lankan, *UR* under reporter

Table 1 reports participant characteristics according to energy reporting status for the total sample and the completers analysis. In the total sample, women who under reported energy intake were younger, had lower physical activity, higher weight and higher BMI at study entry and 36 weeks, and a higher prevalence of smoking at 36 weeks, when compared with women who adequately reported their energy intake. Under reporting of energy intake was more likely among obese women compared with overweight women at both study entry (66.9% vs 33.1%, *p* < 0.001) and 36 weeks gestation (51.4% vs 35.8%, *p* = 0.032). Women who under reported energy intake had poorer diet quality at study entry (Table 1), specifically in relation to consumption of whole fruit, total vegetables, total grains, wholegrains or milk (Additional file1: Table S1). Women who under reported energy intake also had a lower intake of energy (Table 1), macronutrients, fibre, glycaemic load and all micronutrients both at study entry and 36 weeks. These relationships were maintained in the completers analysis for all variables with the exception of physical activity and diet quality (total and wholegrains) at study entry. On completers analysis women who under reported also had lower diet quality in relation to consumption of total fruit (Additional file 1: Table S1).

For the completers analysis, the prevalence of under reporting of energy intake was significantly higher at 36 weeks gestation in comparison to early pregnancy (*p* < 0.001). There was also a pattern of women changing their energy reporting status from early to late pregnancy. 30.1% of women were considered under reporters of energy intake at both time points, 41.4% adequate energy reporters at both time points. 7.9% considered under reporters of energy intake at study entry but adequate energy reporters at 36 weeks and 20.6% were adequate energy reporters at study entry but under reporters of energy intake at 36 weeks. The women who changed from adequate to under reporters of energy intake were older than women who were under reporters of energy intake at both time points (30.6 ± 5.1 vs 28.6 ± 5.1 years, *p* = 0.012), and of higher weight (87.0 ± 16.1 vs 81.5 ± 13.4 kg, *p* = 0.026) and BMI (32.6 ± 5.6 vs 30.0 ± 4.4 kg/m^2^, *p* = 0.001) than those who were adequate energy reporters at both time points.

### Gestational weight gain by energy misreporting status

For the completers analysis, the mean gestational weight gain was 9.8 ± 5.7 kg. 44.3% of women gained above, 21.2% gained below and 34.5% gained within the Institute of Medicine gestational weight gain recommendations. There was no association between under reporting of energy intake and gestational weight gain (*p* = 0.536), or the proportion of women achieving weight gain within the Institute of Medicine recommendations (*p* = 0.444). However, there was a significant time-by-energy reporter status interaction for the change in energy intake across pregnancy (*p* < 0.001). Women who under reported energy intake had a reduction (− 1087.2 ± 2271.9 kJ, *p* < 0.001) and adequate reporters had an increase (649.3 ± 3209.9 kJ, *p* < 0.001) in energy intake from trial entry to 36 weeks.

### Characteristics of women who under reported energy intake

For the completers analysis there were no differences in the risk of depression or anxiety symptoms, dieting behaviour or BMI estimation at either study entry or 36 weeks for women who did or did not under report energy intake (Table 2). While energy reporting status was not associated with body image discrepancy score at study entry, women who under reported energy intake were more likely to report greater dissatisfaction at 36 weeks gestation. Women who under reported their energy intake at 36 weeks were also more likely to report a history of multiple dieting attempts in the previous 12 months, when compared with women who reported an adequate energy intake. Women who under reported their energy intake were less likely to also report dissatisfaction with their weight and shape at both study entry and 36 weeks when compared with women who adequately reported their energy intake.

**Table 2:** Risk of depression, anxiety symptoms, dieting behaviour and weight and shape satisfaction according to energy reporting status

Outcomes		10–20 weeks (completers, *n* = 497)			36 weeks (*n* = 504)		
		UR	AR	*P*	UR	AR	*P*
Risk of depression	mean ± SD	5.4 ± 4.5	5.6 ± 4.2	0.554	5.2 ± 4.6	5.8 ± 4.7	0.175
	Yes	15 (8.6%)	21 (7.3%)	0.613	16 (6.4%)	28 (11.1%)	0.064
	No	159 (91.4%)	266 (92.7%)		234 (93.6%)	225 (88.9%)	
Anxiety symptoms	mean ± SD	10.2 ± 3.4	10.4 ± 3.8	0.530	10.0 ± 3.4	10.5 ± 3.6	0.164
	Yes	24 (12..8%)	40 (13.0%)	0.856	28 (11.2%)	32 (12.7%)	0.593
	No	163 (87.2%)	268 (87.0%)		223 (88.8%)	220 (87.3%)	
Dieting behaviour (times in previous year)	Never	10 (17.9%)	32 (31.7%)	0.164	13 (16.5%)	30 (36.1%)	0.013
	1–10 times	40 (71.4%)	61 (59.8%)		56 (70.9%)	47 (56.6%)	
	> 10 times	6 (10.7%)	9 (8.8%)		10 (12.7%)	6 (7.2%)	
BMI estimate	Underestimate	24 (42.9%)	47 (45.6%)	0.931	38 (48.1%)	40 (47.6%)	0.579
	Correctly estimate	27 (48.2%)	48 (46.6%)		33 (41.8%)	39 (46.4%)	
	Overestimate	5 (8.9%)	8 (7.8%)		8 (10.1%)	5 (6.0%)	
Discrepancy score	mean ± SD	4.1 ± 0.7	3.9 ± 0.6	0. 115	4.1 ± 0.7	3.8 ± 0.6	0.034
Weight satisfaction	Not satisfied	25 (44.6%)	69 (67.0%)	0.006	39 (49.4%)	57 (67.9%)	0.016
	Satisfied	31 (55.4%)	34 (33.0%)		40 (50.6%)	27 (32.1%)	
Shape satisfaction	Not satisfied	25 (44.6%)	71 (68.9%)	0.003	41 (51.9%)	58 (69.0%)	0.025
	Satisfied	31 (55.4%)	32 (31.1%)		38 (48.1%)	26 (31.0%)	

Data are presented as mean ± SD or proportions and were analysed by independent t test or chi squared tests

Over reporters were excluded from analysis

*AR* Adequate reporter, *BMI* Body mass index, *SD* Standard deviation, *UR* Under reporter

The independent contribution of demographic, anthropometric and psychosocial factors to under reporting of energy intake at study entry or 36 weeks is reported in Table 3 for the completers analysis. At study entry, higher social disadvantage and BMI were the only significant independent predictors of under reporting of energy intake. At 36 weeks, higher social disadvantage, higher BMI and greater dieting behaviour were all significantly predictive of under reporting of energy intake (Table 3). Women identified to be at a higher risk of depression were less likely to under report energy intake.

**Table 3:** Logistic regression of under reporter status at trial entry or 36 weeks

Outcomes		10–20 weeks			36 weeks		
		OR	95% CI	*P*	OR	95% CI	*P*
SEIFA	1	1	–	–	1	–	–
	2	0.56	0.20, 1.55	0.262	0.19	0.06, 0.60	0.003
	3	0.45	0.13, 1.59	0.215	0.15	0.04, 0.56	0.005
	4	0.25	0.07, 0.87	0.029	0.16	0.05, 0.58	0.005
	5	0.34	0.09, 1.29	0.113	0.15	0.04, 0.59	0.007
BMI (kg/m^2^)	25–29.99	1	–	–	1	–	–
	30–34.99	1.09	0.43, 2.78	0.850	2.07	0.58, 7.46	0.265
	35–39.99	1.61	0.52, 4.96	0.405	2.60	0.58, 11.66	0.212
	≤40	23.10	2.44, 218.6	0.006	10.27	1.75, 60.30	0.010
Age (years)		1.01	0.93, 1.09	0.786			
Smoking	No	1	–	–			
	Yes	1.57	0.21, 11.7	0.659			
Weight satisfaction	Satisfied	1	–	–	1		
	Not satisfied	0.49	0.16, 1.48	0.207	0.38	0.11, 1.21	0.101
Shape satisfaction	Satisfied	1	–	–	1		
	Not satisfied	0.87	0.29, 2.58	0.806	1.35	0.42, 4.33	0.611
BMI estimate	Correctly estimate	1	–	–			
	Under estimate	0.80	0.36, 1.77	0.576			
	Over estimate	0.68	0.16, 2.82	0.595			
Dieting behaviour	Never				1	–	–
	1–10 times				2.38	1.00, 5.65	0.051
	> 10 times				4.52	1.11, 18.29	0.035
Discrepancy score					1.06	0.55, 2.03	0.864
Risk of depression	No				1	–	–
	Yes				0.20	0.04, 0.93	0.040

Data were analysed by logistic regression

Over reporters were excluded from analysis

*BMI* Body mass index, *CI* Confidence interval, *OR* Odds ratio, *SEIFA* Socioeconomic indices for areas disadvantage score

## Discussion

We report for the first time the association of behavioural and psychological factors with energy misreporting status in a large population of overweight or obese women across pregnancy. At 10–36 weeks gestation under reporting of energy intake was present in up to 50% of women and was independently related to BMI, SEIFA, prior dieting behaviour and depression. The level of under reporting was higher in late pregnancy in comparison to early pregnancy. Diet quality was lower among women who under reported energy intake. We reported a low prevalence of women who over reported intake (0.2–2.3%), in contrast to other reports of up to 12% during pregnancy [11, 16].

As previously reported, under reporting of energy intake was present in a third of overweight or obese women at study entry [16, 37]. As not all overweight and obese women misreport energy intake it is important to identify characteristics that may be predictive of misreporting status. Under reporting of energy intake was more common among obese women compared with overweight women [11, 16, 37] consistent with general population data [2]. The association of low socioeconomic status or education with under reporting of energy intake in the general population or in pregnancy has also been previously reported, and may reflect poor literacy skills [2, 37]. Alternatively, a positive association has also been reported between education and under reporting [43] which may reflect those with a greater knowledge about healthy eating being more prone to selective misreporting. Women may also experience pressure and guilt in relation to achieving an optimal diet during pregnancy [44] which increases as pregnancy progresses and may be compounded by socioeconomic status reflecting poorer health literacy and differences in knowledge and attitudes towards nutrition [45].

We report a range of behavioural or psychological factors that are independently associated with under reporting of energy intake during pregnancy. Specifically, we have identified an independent association between risk of depression and a lower prevalence of under reporting of energy intake in late pregnancy. This is in contrast to prior reports, in which there was no association between risk of depression and low energy reporting, but positive associations with high energy reporting in the second trimester of pregnancy [16]. We found no association between symptoms of anxiety and energy misreporting, which is in contrast to reports from the general population [46]. Increased dieting behaviour prior to pregnancy, greater body dissatisfaction and less dissatisfaction with weight or shape were all associated with a higher level of under reporting of energy intake, in keeping with general population data [4, 47]. However, weight and shape satisfaction associations were attenuated in the logistic regression models indicating a likely indirect relationship between body image and to energy reporting through its relationship with BMI.

There is limited and conflicting literature assessing changes in energy reporting status across pregnancy. A lower level of under reporting of energy intake later in pregnancy assessed by 24-h recall was reported in *n* = 490 pregnant Indonesian women [17]. However, others have reported a higher level of under reporting of energy intake later in pregnancy in 12 women, in the only study utilising the gold-standard assessment of energy expenditure of doubly labelled water [18] consistent with our results presented here. The reason for the higher under reporting occurring at week 36 in our current study is unclear. It was not possible to identify characteristics of women who modified their energy reporting status across pregnancy due to the small event number with the exception of age and BMI. It may be related to factors such as increased time pressures or the effect of weight increasing as pregnancy progresses. We have also previously reported a reduction in depression across pregnancy in this cohort [23]. Given that we reported higher depression was related to lower under reporting on logistic regression analysis, the higher under reporting may be a reflection of this previously observed lower level of depression. While under reporting of energy intake commonly reflects inadequate reporting of dietary intake, it may also reflect an actual restriction in food intake. This may be particularly relevant in overweight or obese women during pregnancy as an attempt to prevent excess weight gain [48]. However, while women who under reported their energy intake at 36 weeks were more likely to report a history of multiple dieting attempts in the previous 12 months, we did not assess if women perceived themselves to be under any dietary restraint during pregnancy itself. In early pregnancy, under reporting of energy intake may also reflect dietary intake secondary to nausea or food aversions. We didnot specifically measure these issues as many women attended the trial entry visit in the second trimester where some of these symptoms may have abated. As these symptoms may affect a large proportion of women (up to 72% in a Norwegian cohort study of *n* = 51,675 women) [49], it is crucial to report this in future research assessing dietary misreporting during pregnancy.

We also identified that women who under report energy intake have a subtly poorer diet quality, largely reflecting reduced core food group intake, as has previously been reported [37]. This is in contrast to data from the general population where under reporters of energy intake have improved diet quality [4], more optimal dietary intake by dietary pattern analysis, [50] or micronutrient densities [2, 7], a lower proportional intake of fat, [4] and lower consumption of non-core foods [4]. Our study comprised only women who were overweight or obese and it is possible that this precluded an accurate assessment of changes in dietary intake across all energy reporting groups. Alternatively, if low energy reporting in pregnancy reflects an actual reduction in food intake then a subset of overweight and obese pregnant women are limiting their dietary intake, which in turn potentially impacts their diet quality.

Our study has focused on women who were overweight and obese, which limits therefore the generalisability of our results. We note that the self-reporting of dietary intake data is associated with a degree of inherent measurement errors [51]. In particular, the use of FFQ is associated with a higher level of under energy reporting of intake, when compared with other tools of dietary assessment, such as multiple 24-h recalls. [3]. This may reflect a number of underlying factors including measurement error relating to recall bias, categorisation of portion size and food items lists that may not reflect a changing and diverse food supply [52]. However, the use of the FFQ is more practical and feasible for use in a large scale clinical trial such as ours, and for ranking individuals in large epidemiological or clinical trials. We note the different recall periods used for the FFQ (12 months versus 8 weeks). This may result in capturing a combination of pre-pregnancy and pregnancy dietary intake for the trial entry FFQ in comparison to only pregnancy dietary intake for the 36 week questionnaire which may introduce bias into our dietary information. SEIFA is also an estimate of socioeconomic status that may not accurately reflect individual or familial disadvantage. [20] The influence of family income, education or occupation on energy misreporting in pregnancy should be considered in future studies. The strengths of this study include the large sample size, a comprehensive assessment of women’s psychological wellbeing and assessment at two time points across pregnancy.

Our detailed assessment of demographic, behavioural and psychological characteristics has identified factors independently associated with energy misreporting across pregnancy. It is important to identify women who are at high risk of energy intake under reporting across pregnancy, who may be suitable for a more intensive and tailored intervention during pregnancy. Furthermore, if under reporting of energy intake in pregnancy reflects a true reduction in food intake, this also highlights a proportion of overweight and obese pregnant women who may have suboptimal dietary intake, and who warrant specific attention for improving their diet quality and nutrient adequacy.

## Conclusion

Under reporting of energy intake was present in over a third of overweight and obese pregnant women and was higher in late compared to early pregnancy. Characteristics such as BMI, socioeconomic status, past dieting behaviour and risk of depression may aid in identifying women who either require support in accurate recording of food intake or attention for improving diet quality.

## Additional file

Additional file 1: **Table S1.** Dietary intake at trial entry and 36 weeks according to energy reporting status. Supplemental Table describing differences in dietary at trial entry and 36 weeks comparing women who were under reporters or adequate reporters of energy. (DOCX 38 kb)

## Abbreviatons

BMR: Basal metabolic rate
BMI: Body mass index
EPDS: Edinburgh Postnatal Depression Scale
FFQ: Food frequency questionnaires
GI: Glycaemic index
GL: Glycaemic load
HEI: Healthy Eating Index
SEIFA: Socioeconomic index for area of disadvantage score
SQUASH: Short Questionnaire to Assess Health-enhancing physical activity
STAI: Spielberger State-Trait Inventory Self Evaluation Questionnaire

## References

[1] Black AE, Cole TJ (2001). Biased over- or under-reporting is characteristic of individuals whether over time or by different assessment methods. J Am Diet Assoc.

[2] Poslusna K, Ruprich J, de Vries JH, Jakubikova M, van't Veer P (2009). Misreporting of energy and micronutrient intake estimated by food records and 24 hour recalls, control and adjustment methods in practice. Br J Nutr.

[3] Freedman LS, Commins JM, Moler JE, Arab L, Baer DJ, Kipnis V, Midthune D, Moshfegh AJ, Neuhouser ML, Prentice RL (2014). Pooled results from 5 validation studies of dietary self-report instruments using recovery biomarkers for energy and protein intake. Am J Epidemiol.

[4] Lutomski JE, van den Broeck J, Harrington J, Shiely F, Perry IJ (2011). Sociodemographic, lifestyle, mental health and dietary factors associated with direction of misreporting of energy intake. Public Health Nutr.

[5] Scagliusi FB, Polacow VO, Artioli GG, Benatti FB, Lancha AH, Jr.: Selective underreporting of energy intake in women: magnitude, determinants, and effect of training. J Am Diet Assoc 2003, 103(10):1306–1313. 10.1016/s0002-8223(03)01074-5 14520248

[6] Mela DJ, Aaron JI (1997). Honest but invalid: what subjects say about recording their food intake. J Am Diet Assoc.

[7] Rosell MS, Hellenius ML, de Faire UH, Johansson GK (2003). Associations between diet and the metabolic syndrome vary with the validity of dietary intake data. Am J Clin Nutr.

[8] Mendez MA, Wynter S, Wilks R, Forrester T (2004). Under- and overreporting of energy is related to obesity, lifestyle factors and food group intakes in Jamaican adults. Public Health Nutr.

[9] Dyer JS, Rosenfield CR (2011). Metabolic imprinting by prenatal, perinatal**,** and Postnatal Overnutrition. Semin Reprod Endocrinol.

[10] Kind KL, Moore VM, Davies MJ (2006). Diet around conception and during pregnancy--effects on fetal and neonatal outcomes. Reprod BioMed Online.

[11] Mullaney L, O'Higgins AC, Cawley S, Doolan A, McCartney D, Turner MJ (2014). An estimation of periconceptional under-reporting of dietary energy intake. Journal of public health.

[12] Dodd JM, Grivell RM, Ngyuen MA, Chan A, Robinson JS (2011). Maternal and perinatal health outcomes by maternal body mass index category. ANZJOG.

[13] Catalano PM, Farrell K, Thomas A, Huston-Presley L, Mencin P, de Mouzon SH, Amini SB (2009). Perinatal risk factors for childhood obesity and metabolic dysregulation. Am J Clin Nutr.

[14] Hochner H, Friedlander Y, Calderon-Margalit R, Meiner V, Sagy Y, Avgil-Tsadok M, Burger A, Savitsky B, Siscovick DS, Manor O (2012). Associations of maternal prepregnancy body mass index and gestational weight gain with adult offspring cardiometabolic risk factors: the Jerusalem perinatal family follow-up study. Circulation.

[15] Kretsch MJ, Fong AK, Green MW (1999). Behavioral and body size correlates of energy intake underreporting by obese and normal-weight women. J Am Diet Assoc.

[16] Nowicki E, Siega-Riz AM, Herring A, He K, Stuebe A, Olshan A (2011). Predictors of measurement error in energy intake during pregnancy. Am J Epidemiol.

[17] Winkvist A, Persson V, Hartini TN (2002). Underreporting of energy intake is less common among pregnant women in Indonesia. Public Health Nutr.

[18] Goldberg GR, Prentice AM, Coward WA, Davies HL, Murgatroyd PR, Wensing C, Black AE, Harding M, Sawyer M (1993). Longitudinal assessment of energy expenditure in pregnancy by the doubly labeled water method. Am J Clin Nutr.

[19] Dodd JM, Turnbull D, McPhee AJ, Deussen AR, Grivell RM, Yelland LN, Crowther CA, Wittert G, Owens JA, Robinson JS (2014). Antenatal lifestyle advice for women who are overweight or obese: LIMIT randomised trial. BMJ.

[20] Australian Bureau of Statistics. Socio-Economic Indexes for Areas (SEIFA) - Technical Paper 2006, vol. 2008. Canberra: Australian Bureau of Statistics.

[21] WHO Obesity and overweight fact sheet (number 311; March 2011): WHO; http://www.who.int/mediacentre/factsheets/fs311/en/index.html.

[22] National Research Council Institute of Medicine. In: Rasmussen KM, Yaktine AL, editors. Weight gain during pregnancy: reexamining the guidelines. Washington D.C; 2009. 20669500

[23] Dodd JM, Newman A, Moran LJ, Deussen AR, Grivell RM, Yelland LN, Crowther CA, McPhee AJ, Wittert G, Owens JA (2016). The effect of antenatal dietary and lifestyle advice for women who are overweight or obese on emotional well-being: the LIMIT randomized trial. Acta Obstet Gynecol Scand.

[24] Cox JL, Holden JM, Sagovsky R (1987). Detection of postnatal depression - development of the 10 item Edinburgh postnatal depression scale (EDPS). Brit J Psych.

[25] Marteau TM, Bekker H (1992). The development of a six item form of the state scale of the Spielberger state trait anxiety inventory (STAI). Br J Clin Psychol.

[26] Spielberger CD. The state-trait anxiety inventory: consulting psychologists press, Inc; 1970.

[27] Bulik CM, Wade TD, Heath AC, Martin NG, Stunkard AJ, Eaves LJ (2001). Relating body mass index to figural stimuli: population-based normative data for Caucasians. Int J Obes Relat Metab Disord.

[28] Sui Z, Turnbull D, Dodd J (2013). Effect of body image on gestational weight gain in overweight and obese women. Women and birth : journal of the Australian College of Midwives.

[29] Willett WC, Reynolds RD, Cottrell-Hoehner S, Sampson L, Browne ML (1987). Validation of a semi-quantitative food frequency questionnaire: comparison with a 1-year diet record. J Am Diet Assoc.

[30] Fawzi WW, Rifas-Shiman SL, Rich-Edwards JW, Willett WC, Gillman MW (2004). Calibration of a semi-quantitative food frequency questionnaire in early pregnancy. Ann Epidemiol.

[31] Rumbold AR, Crowther CA, Haslam RR, Dekker GA, Robinson JS, Group AS (2006). Vitamins C and E and the risks of preeclampsia and perinatal complications. N Engl J Med.

[32] NUTTAB (2010). Australian food composition tables.

[33] Atkinson FS, Foster-Powell K, Brand-Miller JC (2008). International tables of glycemic index and glycemic load values: 2008. Diabetes Care.

[34] Louie JC, Flood V, Turner N, Everingham C, Gwynn J (2011). Methodology for adding glycemic index values to 24-hour recalls. Nutrition.

[35] Dodd JM, Cramp C, Sui Z, Yelland LN, Deussen AR, Grivell RM, Moran LJ, Crowther CA, Turnbull D, McPhee AJ (2014). The effects of antenatal dietary and lifestyle advice for women who are overweight or obese on maternal diet and physical activity: the LIMIT randomised trial. BMC Med.

[36] Schofield WN (1985). Predicting basal metabolic rate, new standards and review of previous work. Human nutrition Clinical nutrition.

[37] McGowan CA, McAuliffe FM (2012). Maternal nutrient intakes and levels of energy underreporting during early pregnancy. Eur J Clin Nutr.

[38] Horan MK, McGowan CA, Gibney ER, Byrne J, Donnelly JM, McAuliffe FM. Maternal nutrition and Glycaemic index during pregnancy impacts on offspring adiposity at 6 months of age--analysis from the ROLO randomised controlled trial. Nutrients. 2016;8(1) 10.3390/nu8010007 PMC4728621 26742066

[39] NHMRC (2005). Nutrient reference values for Australia and New Zealand. Canberra: Australian government publishing service.

[40] Lindsay KL, Heneghan C, McNulty B, Brennan L, McAuliffe FM (2015). Lifestyle and dietary habits of an obese pregnant cohort. Matern Child Health J.

[41] Goldberg GR, Black AE, Jebb SA, Cole TJ, Murgatroyd PR, Coward WA, Prentice AM (1991). Critical evaluation of energy intake data using fundamental principles of energy physiology: 1. Derivation of cut-off limits to identify under-recording. Eur J Clin Nutr.

[42] Lumley T, Diehr P, Emerson S, Chen L (2002). The importance of the normality assumption in large public health data sets. Annu Rev Public Health.

[43] Yannakoulia M, Panagiotakos DB, Pitsavos C, Bathrellou E, Chrysohoou C, Skoumas Y, Stefanadis C (2007). Low energy reporting related to lifestyle, clinical, and psychosocial factors in a randomly selected population sample of Greek adults: the ATTICA study. J Am Coll Nutr.

[44] Wennberg AL, Lundqvist A, Hogberg U, Sandstrom H, Hamberg K (2013). Women's experiences of dietary advice and dietary changes during pregnancy. Midwifery.

[45] Inglis V, Ball K, Crawford D (2005). Why do women of low socioeconomic status have poorer dietary behaviours than women of higher socioeconomic status? A qualitative exploration. Appetite.

[46] Taren DL, Tobar M, Hill A, Howell W, Shisslak C, Bell I, Ritenbaugh C (1999). The association of energy intake bias with psychological scores of women. Eur J Clin Nutr.

[47] Rennie KL, Siervo M, Jebb SA (2006). Can self-reported dieting and dietary restraint identify underreporters of energy intake in dietary surveys?. J Am Diet Assoc.

[48] Storti KL, Arena VC, Barmada MM, Bunker CH, Hanson RL, Laston SL, Yeh JL, Zmuda JM, Howard BV, Kriska AM (2009). Physical activity levels in American-Indian adults**:** the Strong Heart Family Study. Am J Prev Med.

[49] Chortatos A, Haugen M, Iversen PO, Vikanes A, Magnus P, Veierod MB (2013). Nausea and vomiting in pregnancy: associations with maternal gestational diet and lifestyle factors in the Norwegian mother and child cohort study. BJOG.

[50] Scagliusi FB, Ferriolli E, Pfrimer K, Laureano C, Cunha CS, Gualano B, Lourenco B, Lancha AH (2008). Under-reporting of energy intake is more prevalent in a healthy dietary pattern cluster. Br J Nutr.

[51] Subar AF, Freedman LS, Tooze JA, Kirkpatrick SI, Boushey C, Neuhouser ML, Thompson FE, Potischman N, Guenther PM, Tarasuk V (2015). Addressing current criticism regarding the value of self-report dietary data. J Nutr.

[52] Prentice RL, Mossavar-Rahmani Y, Huang Y, Van Horn L, Beresford SA, Caan B, Tinker L, Schoeller D, Bingham S, Eaton CB (2011). Evaluation and comparison of food records, recalls, and frequencies for energy and protein assessment by using recovery biomarkers. Am J Epidemiol.

