# Supplementary material for: The characterisation of overweight and obese women who are under reporting energy intake during pregnancy
Source: BMC Pregnancy Childbirth. 2018 Jun 1;18:204. doi: 10.1186/s12884-018-1826-x (PMC5984749; doi:10.1186/s12884-018-1826-x)
Supplement: Supplementary file 1 — Table S1. Dietary intake at trial entry and 36 weeks according to energy reporting status. Supplemental Table describing differences in dietary at trial entry and 36 weeks comparing women who were under reporters or adequate reporters of energy. (DOCX 38 kb) [file 12884_2018_1826_MOESM1_ESM.docx]

**Table S1: Dietary intake at trial entry and 36 weeks according to energy reporting status**

| Outcomes | | Trial entry | | P AR vs UR Trial entry | 36 weeks | | P AR vs UR 36 weeks |
| --- | --- | --- | --- | --- | --- | --- | --- |
|  |  | UR  N=188 | AR  N=309 |  | UR  N=251 | AR  N=253 |  |
| Macronutrients | Carbohydrate (g) | 165.1±38.2 | 280.9±72.2 | <0.001 | 173.4±45.1 | 307.6±77.6 | <0.001 |
|  | Carbohydrate (%) | 45.8±6.4 | 46.4±5.3 | 0.355 | 46.3±6.4 | 47.1±5.0 | 0.110 |
|  | Protein (g) | 68.8±18.0 | 109.9±27.2 | <0.001 | 70.6±19.0 | 114.7±26.6 | <0.001 |
|  | Protein (%) | 23.8±4.1 | 22.8±3.7 | 0.007 | 23.7±4.4 | 22.2±3.6 | <0.001 |
|  | Fat (g) | 43.5±12.0 | 73.1±19.6 | <0.001 | 45.9±12.5 | 80.8±20.2 | <0.001 |
|  | Fat (%) | 27.8±4.8 | 28.0±4.2 | 0.626 | 28.4±4.7 | 28.7±3.9 | 0.310 |
|  | Saturated fat (g) | 17.6±5.6 | 29.7±9.4 | <0.001 | 19.1±6.0 | 33.5±9.6 | <0.001 |
|  | Saturated fat (%) | 11.2±2.5 | 11.4±2.4 | 0.481 | 11.8±2.6 | 11.9±2.4 | 0.465 |
|  | MUFA (g) | 15.1±4.5 | 25.2±7.3 | <0.001 | 15.8±4.7 | 27.8±7.6 | <0.001 |
|  | MUFA (%) | 9.7±2.1 | 9.6±1.7 | 0.982 | 9.8±2.0 | 9.9±1.7 | 0.439 |
|  | PUFA (g) | 5.9±1.8 | 10.2±3.2 | <0.001 | 6.0±1.8 | 11.1±3.5 | <0.001 |
|  | PUFA (%) | 3.8±0.9 | 3.9±0.8 | 0.088 | 3.7±0.8 | 3.9±0.8 | 0.002 |
|  | Fibre (g) | 21.3±7.9 | 36.2±11.8 | <0.001 | 21.8±7.7 | 37.7±13.2 | <0.001 |
|  | Alcohol (g) | 1.9±5.2 | 3.4±8.1 | 0.023 | 0.20±0.98 | 0.33±1.22 | 0.219 |
| Micronutrients | Sodium (mg) | 1757.0±640.6 | 3017.8±1035.9 | <0.001 | 1835.5±639.9 | 3221.9±948.9 | <0.001 |
|  | Calcium (mg) | 648.9±266.0 | 1112.7±436.6 | <0.001 | 724.2±272.7 | 1227.2±386.7 | <0.001 |
|  | Iron (mg) | 9.3±2.7 | 15.4±4.1 | <0.001 | 9.7±2.8 | 16.7±4.8 | <0.001 |
|  | Zinc (mg) | 8.3±2.2 | 13.2±3.2 | <0.001 | 8.5±2.3 | 13.9±3.4 | <0.001 |
|  | Magnesium (mg) | 232.2±62.6 | 391.2±102.3 | <0.001 | 243.2±66.9 | 411.6±109.1 | <0.001 |
|  | Phosphorus (mg) | 1096.8±272.4 | 1813.6±458.4 | <0.001 | 1173.7±309.2 | 1955.8±444.1 | <0.001 |
|  | Potassium (mg) | 2457.8±676.2 | 4023.6±1044.7 | <0.001 | 2569.4±720.3 | 4218.7±1071.3 | <0.001 |
|  | Iodine (g) | 136.9±70.9 | 232.7±100.3 | <0.001 | 148.9±66.3 | 257.2±98.3 | <0.001 |
|  | Vitamin A **(g) | 988.7±596.2 | 1579.9±773.6 | <0.001 | 983.9±491.9 | 1619.9±966.4 | <0.001 |
|  | Retinol (g) | 247.2±323.7 | 348.9±244.0 | 0.001 | 228.2±154.2 | 458.8±582.9 | <0.001 |
|  | Thiamin (mg) | 1.0±0.3 | 1.7±0.5 | <0.001 | 1.1±0.34 | 1.9±0.63 | <0.001 |
|  | Riboflavin (mg) | 1.5±0.6 | 2.5±0.8 | <0.001 | 1.7±0.59 | 2.8±0.84 | <0.001 |
|  | Niacin (mg) | 16.3±4.5 | 25.1±6.5 | <0.001 | 16.7±5.0 | 26.0±7.0 | <0.001 |
|  | Vitamin C (mg) | 101.8±68.4 | 163.7±87.2 | <0.001 | 95.8±67.7 | 163.2±90.8 | <0.001 |
|  | Vitamin E (mg) | 4.9±1.5 | 8.4±2.8 | <0.001 | 5.0±1.7 | 8.9±2.5 | <0.001 |
|  | Total folate (g) | 347.2±121.1 | 593.5±192.2 | <0.001 | 367.1±120.3 | 466.3±163.9 | <0.001 |
|  | Food folate (g) | 263.3±101.8 | 447.9±148.0 | <0.001 | 277.0±100.0 | 466.3±163.9 | <0.001 |
| Other dietary factors | Glycemic index | 50.5±3.9 | 50.5±3.6 | 0.949 | 50.3±3.9 | 50.3±3.4 | 0.860 |
|  | Glycemic load | 83.7±21.5 | 142.3±39.5 | <0.001 | 87.4±25.1 | 155.3±42.4 | <0.001 |
|  | Caffeine (mg) | 92.0±116.6 | 160.2±164.0 | <0.001 | 104.7±124.8 | 142.5±133.7 | 0.001 |
| Diet quality components | HEI total fruit | 4.3±1.2 | 4.5±1.0 | 0.012 | 4.2±1.3 | 4.4±1.1 | 0.090 |
|  | HEI whole fruit | 4.3±1.3 | 4.7±0.9 | 0.002 | 4.2±1.5 | 4.6±1.0 | 0.005 |
|  | HEI total veg | 4.8±0.7 | 4.9±0.5 | 0.033 | 4.8±0.6 | 4.8±0.6 | 0.674 |
|  | HEI dark veg | 4.6±0.9 | 4.8±0.8 | 0.070 | 4.7±0.9 | 4.7±0.9 | 0.728 |
|  | HEI total grain | 3.8±0.9 | 4.0±0.9 | 0.041 | 3.8±1.0 | 4.1±0.9 | <0.001 |
|  | HEI wholegrain | 0.64±0.82 | 0.79±0.85 | 0.065 | 0.55±0.77 | 0.85±0.99 | <0.001 |
|  | HEI milk | 6.6±3.1 | 7.2±2.6 | 0.015 | 7.4±2.7 | 7.5±2.6 | 0.625 |
|  | HEI meat bean | 9.6±1.1 | 9.5±1.2 | 0.841 | 9.4±1.5 | 9.4±1.3 | 0.906 |
|  | HEI oil | 5.3±3.5 | 5.5±3.1 | 0.572 | 4.9±3.4 | 5.1±3.2 | 0.521 |
|  | HEI sat fat | 5.4±3.0 | 5.4±3.0 | 0.773 | 4.8±3.1 | 4.6±2.9 | 0.492 |
|  | HEI sodium | 6.4±2.5 | 6.1±2.4 | 0.174 | 6.3±2.4 | 6.1±2.4 | 0.239 |
|  | HEI SOFAAS | 16.4±3.6 | 16.0±3.4 | 0.243 | 15.7±3.6 | 15.4±3.2 | 0.307 |

Over reporters were excluded from analysis

Data are presented as mean±SD and were analysed by independent t test

** Total vitamin A equivalents

AR: Adequate reporter; HEI: Healthy eating index; MUFA: Monounsaturated fat; PUFA: Polyunsaturated fat; SOFAAS: solid fats, alcohol and added sugars; UR: Under reporter
